# Farnesoid X receptor functions in cervical cancer via the p14^ARF^-mouse double minute 2-p53 pathway

**DOI:** 10.1007/s11033-022-07201-x

**Published:** 2022-03-28

**Authors:** Xiaohua Huang, Bin Wang, Huimin Shen, Danmei Huang, Ganggang Shi

**Affiliations:** 1 Department of Pediatrics, Second Affiliated Hospital of Shantou University Medical College, Shantou, China; 2 Department of Pharmacology, Shantou University Medical College, Shantou, China; 3 Department of Neurology, Second Affiliated Hospital of Shantou University Medical College, Shantou, China

**Keywords:** FXR, Cervical cancer, p14^ARF^, MDM2, p53

## Abstract

**Background:**

Cervical cancer is the second most common cancer among women living in developing countries. Farnesoid X receptor (FXR) is a member of the nuclear receptor family, which regulates the development and proliferation of cancer. However, the role of and molecular mechanism by which FXR acts in cervical cancer are still unknown.

**Methods and results:**

The relationship between FXR and the proliferation of cervical cancer cell lines was detected by MTT and colony formation assays. Immunohistochemistry was used to detect the expression of FXR in cervical cancer tissue slides. Western blotting was used to detect the expression of p14^ARF^, mouse double minute 2 (MDM2) and p53 when FXR was overexpressed or siRNA was applied. Western blotting was used to detect the expression of MDM2 and p53 when pifithrin-α (PFT-α) was applied. FXR activation inhibited the proliferation of cervical cancer cell lines. FXR was significantly decreased in cervical squamous cell carcinoma, which was correlated with TNM stage, but not with metastasis. Overexpression of FXR activated the p14^ARF^-MDM2-p53 pathway. As a p53 inhibitor, PFT-α increased MDM2 in Lenti-vector cells, but had no effect on MDM2 in Lenti-FXR cells.

**Conclusions:**

FXR inhibits cervical cancer by upregulating the p14^ARF^-MDM2-p53 pathway. Activation of FXR may be a potential strategy for the treatment of cervical cancer.

**Supplementary Information:**

The online version contains supplementary material available at 10.1007/s11033-022-07201-x.

## Introduction

Cervical cancer is the second most common cancer among women living in developing countries, with 230,000 deaths every year [1] and 570,000 new cases diagnosed in 2018 alone [2]. The treatment includes surgery, radiotherapy and chemotherapy. The side effects of radiotherapy and chemotherapy are obvious, and chemotherapy resistance should be considered. Therefore, it is necessary to find other methods to treat cervical cancer [3].

The nuclear receptor (NR) superfamily is composed of 48 members. After activation by specific ligands such as hormones, vitamins, and lipophilic metabolites, the NR superfamily activates target genes and mediates a wide range of physiological processes, including development, metabolism and reproduction. Farnesoid X receptor (FXR) is a member of the NR family that not only participates in the regulation of bile acid, lipid [4] and glucose metabolism [5] but also plays an important role in tumorigenesis. FXR controls the balance of bile acids and protects the liver from bile acid-induced damage. FXR^−/−^ mice show persistent hypercholesterolemia and spontaneous liver cancer at 12 to 15 months old [6, 7]. In addition to hepatocarcinoma, FXR is also involved in the tumorigenesis of other cancers. FXR agonists such as chenodeoxycholic acid (CDCA) increase the apoptosis of Barrett's esophagus cells and inhibit the occurrence of esophageal cancer [8]. FXR deletion leads to intestinal cancer. Activation of FXR may be a target for the prevention or treatment of colon cancer [9]. FXR also plays a key role in hormone-related tumors. The FXR agonists CDCA and GW4064 induce apoptosis in the breast cancer cell lines MCF-7 and MDA-MB-468 [10]. CDCA inhibits the growth of R2C cells, which indicate that FXR antagonizes the androgen pathway and inhibits the growth of Leydig cells [11]. However, the role of FXR in growth regulation, apoptosis and tumorigenesis is not completely understood, especially in cervical cancer.

Cyclin-dependent kinase inhibitor 2A (CDKN2A) is located on human chromosome 9p21 and encodes two relatively independent proteins, p14^ARF^ and p16^INK4A^. These two proteins are formed by splicing of different mRNA fragments from the same gene and regulate the cell cycle and apoptosis through the p14^ARF^-mouse double minute 2 (MDM2)-p53 and p16^INK4a^-cyclin-dependent kinases 4 and 6 (CDK4/6)-pRb pathways, respectively [12]. As a cell cycle regulator, p14^ARF^ is silenced in many tumors and inhibits cell proliferation in the G1-S and G2-M phases [13, 14]. Overexpression of MDM2 has been shown to exert oncogenic activity and is associated with tumor grade and prognosis [15, 16]. MDM2 is dysregulated in many cancers and exerts oncogenic activity [17] mainly by promoting the nuclear export and degradation of p53 [18] and inhibiting the anticancer effect of p53 [19]. P53, located on chromosome 17p, functions in cell cycle regulation. Inactivation of p53 leads to tumor proliferation and development [20] and plays an important role in cervical cancer.

Despite this extensive background knowledge, the role of FXR and its mechanism in cervical cancer remain unclear. Therefore, this study aims to explore the role and mechanism of FXR in cervical cancer. We demonstrate that FXR inhibits cervical cancer by upregulating the p14^ARF^-MDM2-p53 pathway.

## Methods

### Chemicals

Pifithrin-α (PFT-α, Santa Cruz, USA) was dissolved in dimethyl sulfoxide (DMSO, Sigma, USA) to a final stock concentration of 20 µmol/l.

### Cell culture

The cervical adenocarcinoma cell lines CaSki and HeLa were procured from the American Type Culture Collection (ATCC, USA), and the cervical squamous cell carcinoma cell line SiHa was procured from the Cell Bank of Typical Culture Preservation Committee of the Chinese Academy of Sciences (China). The cell lines were cultured in Dulbecco’s modified Eagle’s medium (DMEM, Life Technologies, USA) supplemented with 10% fetal bovine serum (Biowest, USA) and 100 µg/ml penicillin and streptomycin (Beyotime, China) in a humid atmosphere with 5% CO_2_ at 37 °C. The medium was changed every two days. The cells were identified by morphological identification and short tandem repeat (STR) analysis.

### MTT

CaSki, HeLa and SiHa cells were plated on 96-well plates (3 × 10^3^ cells/well). Different FXR agonists, such as cholic acid (CA, 100 µg/ml) [21], CDCA (50 µmol/l) [22–24] and GW4064 (2 µmol/l) [22], were added and incubated for 24 h, 48 h and 72 h. Subsequently, MTT (5 mg/ml, Sigma, USA) was transferred to each well, and the plates were incubated for another 4 h. Then, DMSO was added to dissolve the formazan crystals. The signal of each plate was measured at 490 nm on a microplate reader (SpectraMax, USA). The cell viability ratio was calculated by the following formula: [A(control) − A(treated)]/A (control) × 100%. A(control) and A(treated) are the average absorbance of three parallel experiments.

### Colony formation assay

Cells were plated in 6-well dishes at 500 cells/well. The cells were treated with CDCA (50 μmol/l) after adhesion and incubated for 2 weeks until the colonies were large enough to be visualized. Then, the cells were washed with PBS, fixed with 4% paraformaldehyde and stained with Giemsa. Clones were counted by Image-Pro Plus 6 software. The colony formation rate was calculated as follows: colony formation rate = (colony numbers/cell inoculation numbers) × 100%.

### Immunohistochemistry

A tissue slide containing 183 tissue samples was purchased from Alenabio (China, Supplementary Table 1). The tissue slide was deparaffinized in xylene and rehydrated in different concentrations of alcohol. After being boiled in citrate buffer at high temperature for antigen retrieval, the slides were incubated with H_2_O_2_ to block endogenous oxidase activity and then blocked with 5% BSA (Boster, USA). The slide was incubated with rabbit anti-FXR primary antibody (Supplementary Table 2) at 4 ℃ overnight. The next day after washing with PBS, the slide was incubated with goat anti-rabbit IgG-HRP (Supplementary Table 2). The slide was examined with an Olympus microscope and analyzed by Image-Pro Plus 6 software.

### Lentivirus-mediated transfection

A lentivirus encoding FXR was transfected into cervical cancer cells, and the medium was changed after 12 h. Because the lentivirus carries the puromycin resistance gene, puromycin was used to screen puromycin-resistant cells with FXR overexpression, which was confirmed by western blot.

### RNA extraction and real-time quantitative PCR

RNA was extracted with TRIzol (Takara Bio, Inc.). After being washed with 75% alcohol, RNA was dissolved in 20 μl DEPC-treated water, and the concentration was determined by spectrophotometry. PrimeScript™ reverse transcriptase (Takara Bio, Inc.) was used for reverse transcription. Real-time quantitative PCR was performed using an ABI 7500 machine with reaction conditions of 95 °C for 5 s and 60 °C for 34 s for 40 cycles. β-Actin acted as a reference. The primer sequences are shown in Supplementary Table 3.

### Western blot

A total of 3 × 10^5^ cells were seeded in 60 cm^2^ culture dishes, washed with cold PBS and lysed with RIPA lysis buffer (Beyotime, China) on ice. The concentration of protein was measured by a Pierce™ Protein BCA assay kit (Thermo, USA) with a microplate reader (SpectraMax, USA) at 562 nm. Twenty micrograms of protein extracts from the cell were resolved by 10–12% SDS-PAGE and transferred to nitrocellulose membranes (Boster, USA). After being blocked at 37 °C for 1 h, the membranes were incubated with the primary antibodies shown in Supplementary Table 2 at 4 °C overnight, followed by incubation with HRP anti-rabbit or HRP anti-mouse IgG (Supplementary Table 2). β-Actin was used as a control.

### Gene silencing by small interfering RNA (siRNA)

CaSki, HeLa and SiHa cells were transiently transfected with siRNA by using Lipofectamine 2000 (Invitrogen, USA). The medium was replaced with fresh medium 6 h after transfection, and protein was extracted 48 h after transfection. Hs-p14^ARF^/MDM2/TP53-siRNA (Supplementary Table 4) was purchased from Biotend Co., Ltd. (China).

## Results

### FXR inhibits the proliferation of cervical cancer cell lines

**Fig. 1:**
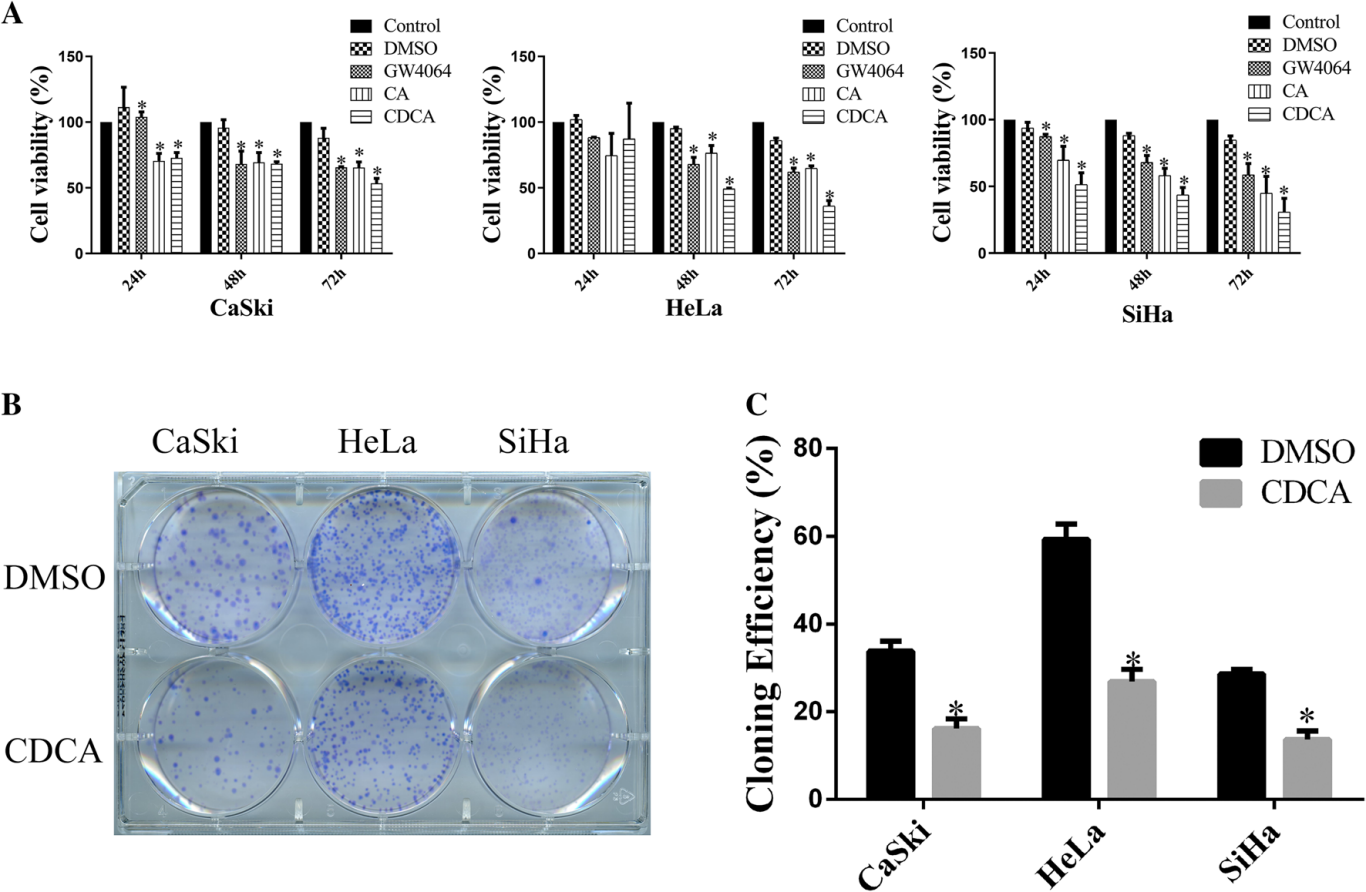
FXR inhibits the proliferation of cervical cancer cell lines

To determine the relationship between FXR and cervical cancer cell lines, MTT and colony formation assays were used to detect the effect of FXR agonists on cervical cancer cell lines. As shown in Fig. 1A, GW4064, CA and CDCA decreased the survival rate of cervical cancer cells compared with DMSO (*p* < 0.05). CDCA is the most effective physiological ligand for FXR [25, 26], as confirmed by the MTT analysis. CDCA had the strongest inhibitory effect on cervical cancer cell lines at 72 h. Therefore, CDCA was chosen as the FXR agonist in the colony formation assay and showed stronger inhibition ability than DMSO in CaSki, HeLa and SiHa cells (Fig. 1B, C, p < 0.05).

### Expression of FXR in cervical cancer tissues

**Fig. 2:**
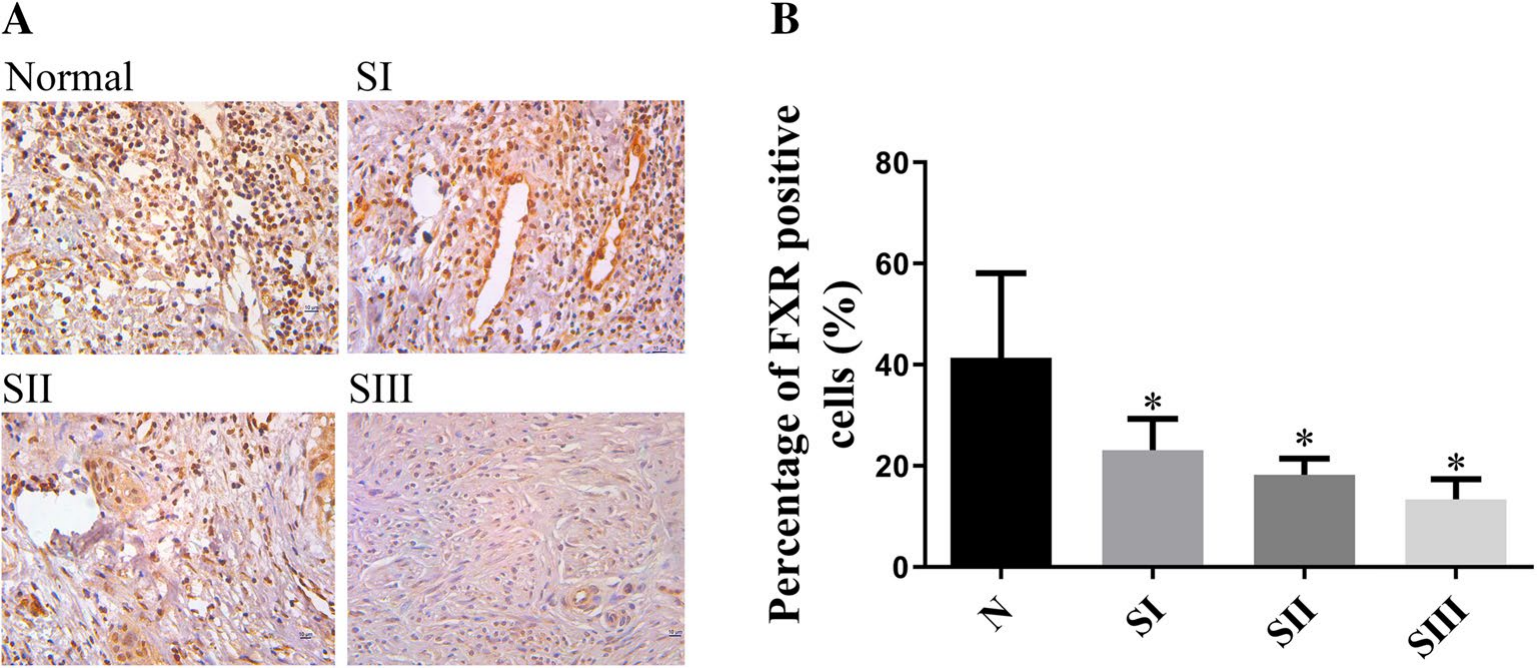
Expression of FXR in cervical cancer tissues

To confirm the relationship between FXR and cervical cancer tissues, the expression of FXR was detected in human normal cervical tissues and cervical cancer tissues to determine whether FXR is involved in cervical cancer. A tissue microarray containing 162 specimens of cervical cancer tissues and 21 specimens of normal cervical tissues was assessed by immunohistochemistry (Supplementary Table 1). As shown in Fig. 2, the FXR protein content in normal cervical tissues was approximately twice as high as that in cervical cancer tissues (*p* < 0.05) and decreased gradually with stage. That is, FXR may play an important role in the occurrence of cervical cancer. FXR was significantly decreased in cervical squamous cell carcinoma and correlated with TNM stage. However, a correlation between FXR and tumor metastasis was not observed.

### Construction of cervical cancer cell lines stably overexpressing FXR

**Fig. 3:**
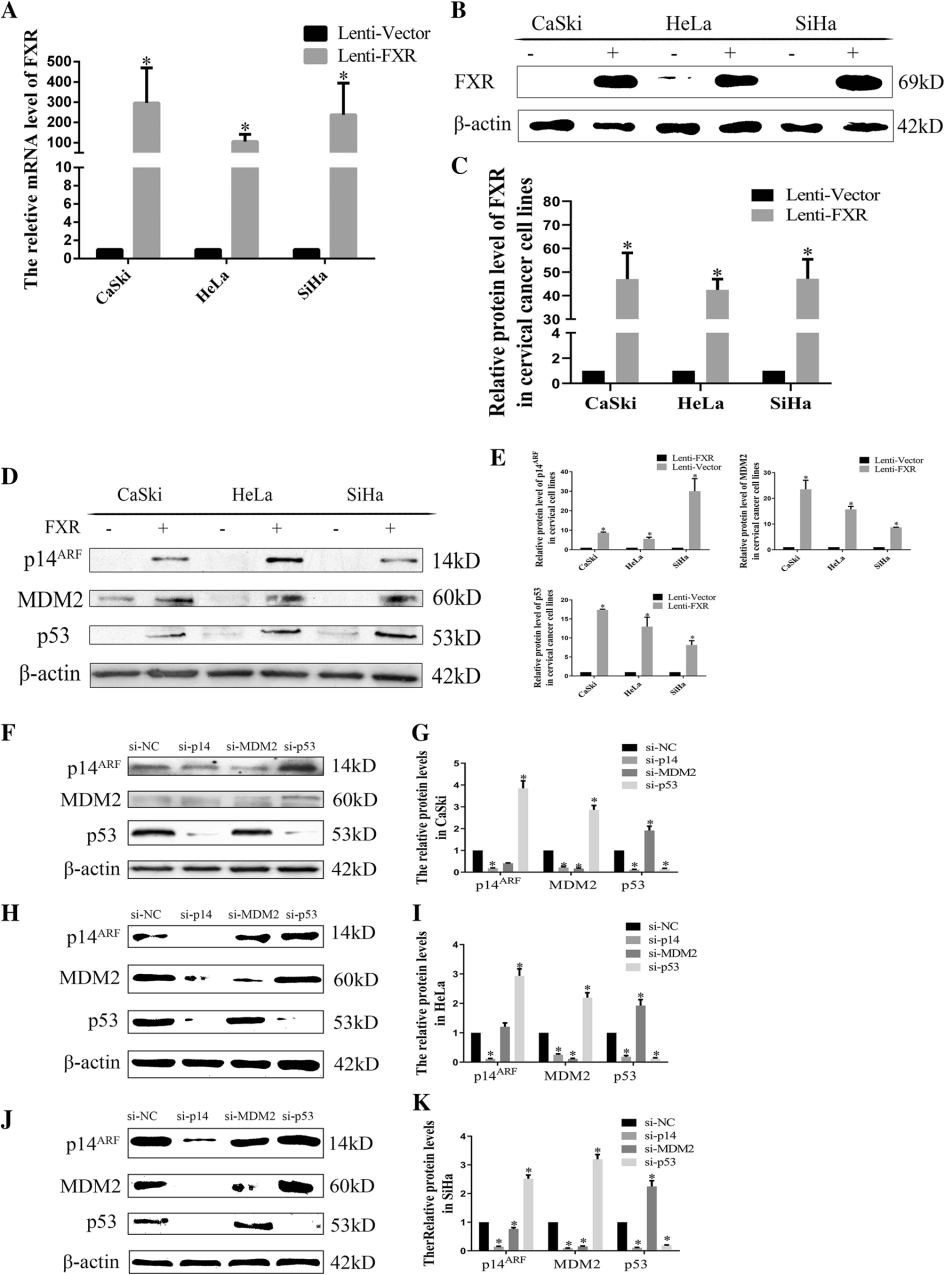
FXR upregulates the p14^ARF^-MDM2-p53 pathway in cervical cancer cell lines

To further study the relationship between FXR and cervical carcinogenesis, FXR-overexpressing cell lines (Lenti-FXR) and the vector control (Lenti-Vector) were constructed by lentivirus transfection. The results showed that the mRNA levels of FXR in the Lenti-FXR groups were 100 times higher than those in the Lenti-Vector groups (Fig. 3A, p < 0.05), while the FXR protein levels in the Lenti-FXR groups were increased by approximately 40 times compared with those in the Lenti-Vector groups (Fig. 3B, C, p < 0.05).

### Overexpression of FXR activates the p14^ARF^-MDM2-p53 pathway

To elucidate the mechanism by which FXR overexpression inhibits the proliferation of cervical cancer cell lines, the levels of protein in the p14^ARF^-MDM2-p53 pathway were detected. Overexpression of FXR increased the levels of protein in the p14^ARF^-MDM2-p53 pathway in CaSki, HeLa and SiHa cell lines compared with those in the Lenti-Vector groups (Fig. 3D, E, p < 0.05).

To study the relationship among members of the p14^ARF^-MDM2-p53 pathway, siRNAs were used to knockdown p14^ARF^, MDM2 or p53 individually, and the levels of the other two proteins were detected by western blot. We found that the protein levels of MDM2 and p53 decreased when p14^ARF^ was knocked down. The protein levels of p14^ARF^ remained unchanged while the protein levels of p53 increased when MDM2 was knocked down. The protein levels of both p14^ARF^ and MDM2 were increased when p53 was knocked down (Fig. 3F–K, p < 0.05).

**Fig. 4:**
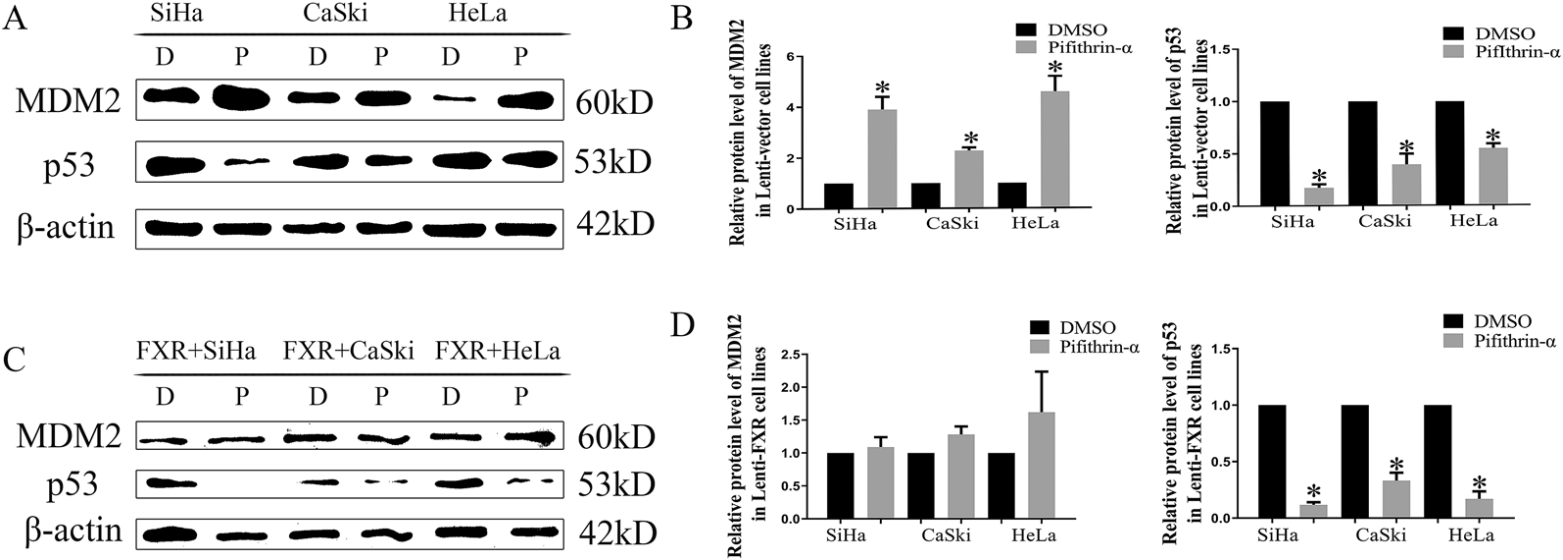
Protein levels of MDM2 in cervical cancer cells with or without PFT-α treatment

To clarify the relationship between MDM2 and p53, PFT-α, a p53 inhibitor, was applied. PFT-α is a small molecule that has been widely used as a specific inhibitor of p53 [27]. In Lenti-vector cells, the protein levels of MDM2 in the PFT-α groups were increased compared with those in the DMSO groups (Fig. 4A, B, p < 0.05). However, there was no significant difference in MDM2 protein levels in Lenti-FXR cells between the PFT-α groups and DMSO groups (Fig. 4C, D, p < 0.05).

## Discussion

In 2018, approximately 570,000 cases of cervical cancer were diagnosed and 311,000 people died of the disease worldwide. Cervical cancer is the fourth most common cancer in women. The number of cervical cancer cases is particularly concerning in developing countries such as China and India [28]. The development of cervical cancer is complicated. Among many factors, human papillomavirus (HPV) is the main pathogenic factor [29]. Many studies have shown that high-risk HPV promotes the occurrence and development of cervical cancer by inhibiting key proteins such as p53 [30]. In fact, less than 10% of people infected with HPV eventually develop cervical cancer, indicating that other factors are involved in the formation and development of cervical cancer.

In this study, according to MTT and colony formation assays, FXR activation inhibited the proliferation of three cervical cancer cell lines. These findings are consistent with the ability of FXR overexpression to inhibit the proliferation of cervical cancer cells, leading to G1 phase arrest [31]. This effect may be related to HPV E6 and E7 proteins, which degrade the tumor suppressors p53 and pRb, resulting in S phase entry without G1 arrest [32]. Resveratrol, a naturally polyphenolic compound found in grapes, peanuts and other plant sources, inhibits the occurrence of cervical cancer by inhibiting the transcription and translation of E6 and E7, promoting cell apoptosis and G1/S arrest [33].

It is well known that estrogen signaling in the tumor microenvironment is related to the tumorigenesis and progression of cervical cancer [34]. FXR may play a role in cervical cancer through estrogen. Activation of FXR has been confirmed to inhibit estrogen signaling in breast cancer, testicular cancer and endometriosis [35]. FXR is a negative regulator of the estrogen-converting aromatase enzyme. The interaction between FXR and estrogen receptor (ER) activated by CDCA induced apoptosis of the positive ER cell line MCF-7 [10], while the negative ER cell line MDA-MB-231 was not sensitive to FXR [36], which indicated that FXR could induce apoptosis of breast cancer cells through the estrogen pathway. The antiproliferative effect of CDCA on Leydig cells was partly due to the inhibition of estrogen-dependent cell growth, which confirmed that the FXR agonist is a negative regulator of aromatase in testis stromal tumor cell lines [11].

CDKN2A, located on human chromosome 9p21, encodes two tumor suppressor genes, p14^ARF^ and p16^INK4A^, which enhance the growth inhibition of p53 and pRb through the p14^ARF^-MDM2-p53 and p16^INK4A^-CDK4/6-pRb pathways, respectively. In contrast, the E6 and E7 oncoproteins of high-risk HPV associated with cervical carcinogenesis promote tumor development by inactivating p53 and pRb [37]. We found that overexpression of FXR activated the p14^ARF^-MDM2-p53 pathway.

As a cell cycle regulator, p14^ARF^ mediates the G1-S/G2-M checkpoints of the cell cycle [38] in a p53-dependent manner, leading to either cell cycle arrest or apoptosis [39]. P14^ARF^ prevents the formation of the MDM2-p53 complex, thus inhibiting the degradation of p53 induced by MDM2. MDM2 and p53 combine in the nucleus and migrate from the nucleus to the cytoplasm, where p53 is ubiquitinated and degraded by ubiquitin ligase E3, which inhibits the activity of p53. p14^ARF^ binds to MDM2, fixes it in the nucleus, and inhibits the effect of MDM2 on p53, thus reducing the ubiquitination of p53, increasing protein synthesis, and causing cell cycle arrest in the G1/S or G2/M phase [40, 41].

In the siRNA assay, we found that the protein levels of MDM2 and p53 were decreased in Lenti-FXR cells when p14^ARF^ was knocked down. On the one hand, p14^ARF^ binds to MDM2 and fixes it to the nucleolus, inhibits the effect of MDM2 on p53, and reduces the ubiquitination of p53 [40, 41]. On the other hand, overexpression of FXR inhibits the binding, translocation to the cytoplasm, and ubiquitination of MDM2 and p53 by forming the SHP-MDM2 complex in the nucleus, thus increasing the expression of MDM2 and p53 [31]. p14^ARF^ is the upstream of MDM2 and p53. Originally, FXR overexpression caused an increase in MDM2 and p53 by inhibiting degradation, which did not require p14^ARF^. In this case, si-p14^ARF^ decreased the expression of MDM2 and p53, probably by regulating their transcription. However, the mechanism of transcription regulation has not been explored, and further research is needed.

The protein levels of p14^ARF^ remained unchanged while the protein levels of p53 increased when MDM2 was knocked down in Lenti-FXR cells. MDM2 and p53 bind in the nucleus and migrate from the nucleus to the cytoplasm by forming the MDM2-p53 complex. p53 is ubiquitinated and degraded by ubiquitin ligase E3, which plays an important role in inhibiting the activity of p53 [40, 41]. In the case of MDM2 knockdown, p53 cannot migrate to the nucleus because ubiquitination and degradation are reduced, so p53 expression is increased.

**Fig. 5:**
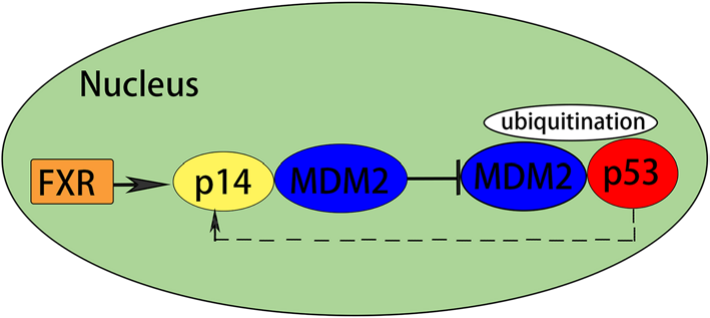
The mechanism of FXR-mediated the p14^ARF^-MDM2-p53 pathway

The protein levels of both p14^ARF^ and MDM2 were increased when p53 was knocked down. p53 induces negative feedback regulation of p14^ARF^. Knockdown of p53 promotes the transcription, translation and protein synthesis of p14^ARF^ (Fig. 5). Increased p14^ARF^ leads to the stabilization of MDM2 because p14^ARF^ directly binds to MDM2 [42].

PFT-α is a p53 inhibitor that blocks p53-dependent transcriptional activity [43]. In Lenti-vector cells, the protein levels of MDM2 in the PFT-α groups were increased, probably because the reduced p53 level decreased the formation of the MDM2-p53 complex and the ubiquitination and degradation of MDM2 [44]. However, MDM2 was not degraded because of the formation of the SHP-MDM2 complex in Lenti-FXR cells [31]. Therefore, there was no significant difference in MDM2 protein levels between the PFT-α groups and DMSO groups. In a previous study, Nutlin-3a was used as an inhibitor of MDM2 and E3 ubiquitin ligases [45]. The results showed that MDM2 and p53 protein levels were increased in Lenti-Vector cells upon the addition of Nutlin-3a because Nutlin-3a suppressed MDM2 binding to p53, thus inhibiting the translocation and ubiquitination of MDM2 and p53, which subsequently increased MDM2 and p53 levels. The expression levels of MDM2 and p53 remained unchanged in Lenti-FXR cells following treatment with Nutlin-3a because overexpression of FXR induced SHP-MDM2 complex formation, thereby reducing MDM2 and p53 binding as well as ubiquitination such that Nutlin-3a’s effects were undetectable [31].

## Conclusion

FXR is significantly decreased in cervical cancer tissues and inhibits the proliferation of cervical cancer cells by inducing apoptosis. In addition, we also demonstrated that FXR increased the contents of p14^ARF^, MDM2 and p53 by activating the p14^ARF^-MDM2-p53 pathway. These findings suggest that FXR agonists represent a potential effective product for the prevention and treatment of cervical cancer.

## Supplementary Information

Below is the link to the electronic supplementary material.

Supplementary file1 (DOCX 16 kb)

## References

[1] Manini I, Montomoli E (2018). Epidemiology and prevention of human papillomavirus. Ann Ig.

[2] Bray F, Ferlay J, Soerjomataram I, Siegel RL, Torre LA, Jemal A (2018). Global cancer statistics 2018: GLOBOCAN estimates of incidence and mortality worldwide for 36 cancers in 185 countries. CA Cancer J Clin.

[3] Ferlay J, Soerjomataram I, Dikshit R, Eser S, Mathers C, Rebelo M, Parkin DM, Forman D, Bray F (2015). Cancer incidence and mortality worldwide: sources, methods and major patterns in GLOBOCAN 2012. Int J Cancer.

[4] Lefebvre P, Cariou B, Lien F, Kuipers F, Staels B (2009). Role of bile acids and bile acid receptors in metabolic regulation. Physiol Rev.

[5] Zhang Y, Lee FY, Barrera G, Lee H, Vales C, Gonzalez FJ, Willson TM, Edwards PA (2006). Activation of the nuclear receptor FXR improves hyperglycemia and hyperlipidemia in diabetic mice. Proc Natl Acad Sci USA.

[6] Yang F, Huang X, Yi T, Yen Y, Moore DD, Huang W (2007). Spontaneous development of liver tumors in the absence of the bile acid receptor Farnesoid X receptor. Cancer Res.

[7] Kim I, Morimura K, Shah Y, Yang Q, Ward JM, Gonzalez FJ (2007). Spontaneous hepatocarcinogenesis in Farnesoid X receptor-null mice. Carcinogenesis.

[8] Matsuzaki J, Suzuki H, Tsugawa H, Watanabe M, Hossain S, Arai E, Saito Y, Sekine S, Akaike T, Kanai Y, Mukaisho K, Auwerx J, Hibi T (2013). Bile acids increase levels of microRNAs 221 and 222, leading to degradation of CDX2 during esophageal carcinogenesis. Gastroenterology.

[9] Maran RR, Thomas A, Roth M, Sheng Z, Esterly N, Pinson D, Gao X, Zhang Y, Ganapathy V, Gonzalez FJ, Guo GL (2009). Farnesoid X receptor deficiency in mice leads to increased intestinal epithelial cell proliferation and tumor development. J Pharmacol Exp Ther.

[10] Swales KE, Korbonits M, Carpenter R, Walsh DT, Warner TD, Bishop-Bailey D (2006). The Farnesoid X receptor is expressed in breast cancer and regulates apoptosis and aromatase expression. Cancer Res.

[11] Catalano S, Malivindi R, Giordano C, Gu G, Panza S, Bonofiglio D, Lanzino M, Sisci D, Panno ML, Ando S (2010). Farnesoid X receptor, through the binding with steroidogenic factor 1-responsive element, inhibits aromatase expression in tumor Leydig cells. J Biol Chem.

[12] Hirao T, Bueno R, Chen CJ, Gordon GJ, Heilig E, Kelsey KT (2002). Alterations of the p16(INK4) locus in human malignant mesothelial tumors. Carcinogenesis.

[13] Yi Y, Shepard A, Kittrell F, Mulac-Jericevic B, Medina D, Said TK (2004). p19ARF determines the balance between normal cell proliferation rate and apoptosis during mammary gland development. Mol Biol Cell.

[14] Eymin B, Gazzeri S, Brambilla C, Brambilla E (2002). Mdm2 overexpression and p14(ARF) inactivation are two mutually exclusive events in primary human lung tumors. Oncogene.

[15] Rayburn E, Zhang R, He J, Wang H (2005). MDM2 and human malignancies: expression, clinical pathology, prognostic markers, and implications for chemotherapy. Curr Cancer Drug Targets.

[16] Karni-Schmidt O, Lokshin M, Prives C (2016). The roles of MDM2 and MDMX in cancer. Annu Rev Pathol.

[17] Wade M, Li YC, Wahl GM (2013). MDM2, MDMX and p53 in oncogenesis and cancer therapy. Nat Rev Cancer.

[18] Marchenko ND, Wolff S, Erster S, Becker K, Moll UM (2007). Monoubiquitylation promotes mitochondrial p53 translocation. EMBO J.

[19] Vaseva AV, Moll UM (2009). The mitochondrial p53 pathway. Biochim Biophys Acta.

[20] Oka K, Suzuki Y, Nakano T (2000). Expression of p27 and p53 in cervical squamous cell carcinoma patients treated with radiotherapy alone: radiotherapeutic effect and prognosis. Cancer.

[21] Schmid A, Schlegel J, Thomalla M, Karrasch T, Schäffler A (2019). Evidence of functional bile acid signaling pathways in adipocytes. Mol Cell Endocrinol.

[22] Li L, Liu H, Peng J, Wang Y, Zhang Y, Dong J, Liu X, Guo D, Jiang Y (2013). Farnesoid X receptor up-regulates expression of lipid transfer inhibitor protein in liver cells and mice. Biochem Biophys Res Commun.

[23] Wang W, Zhan M, Li Q, Chen W, Chu H, Huang Q, Hou Z, Man M, Wang J (2016). FXR agonists enhance the sensitivity of biliary tract cancer cells to cisplatin via SHP dependent inhibition of Bcl-xL expression. OncoTarget.

[24] Rizzo G, Renga B, Mencarelli A, Pellicciari R, Fiorucci S (2005). Role of FXR in regulating bile acid homeostasis and relevance for human diseases. Curr Drug Targets Immune Endocr Metab Disord.

[25] Ding L, Yang L, Wang Z, Huang W (2015). Bile acid nuclear receptor FXR and digestive system diseases. Acta Pharm Sin B.

[26] Li Y, Jadhav K, Zhang Y (2013). Bile acid receptors in non-alcoholic fatty liver disease. Biochem Pharmacol.

[27] Guo J, Tang Q, Wang Q, Sun W, Pu Z, Wang J, Bao Y (2019). Pifithrin-α enhancing anticancer effect of topotecan on p53-expressing cancer cells. Eur J Pharm Sci.

[28] Arbyn M, Weiderpass E, Bruni L, de Sanjosé S, Saraiya M, Ferlay J, Bray F (2020). Estimates of incidence and mortality of cervical cancer in 2018: a worldwide analysis. Lancet Glob Health.

[29] Hu Z, Ma D (2018). The precision prevention and therapy of HPV-related cervical cancer: new concepts and clinical implications. Cancer Med.

[30] de Sanjosé S, Brotons M, Pavón MA (2018). The natural history of human papillomavirus infection. Best Pract Res Clin Obstet Gynaecol.

[31] Huang X, Wang B, Chen R, Zhong S, Gao F, Zhang Y, Niu Y, Li C, Shi G (2021). The nuclear Farnesoid X receptor reduces p53 ubiquitination and inhibits cervical cancer cell proliferation. Front Cell Dev Biol.

[32] Szymonowicz KA, Chen J (2020). Biological and clinical aspects of HPV-related cancers. Cancer Biol Med.

[33] Sun X, Fu P, Xie L, Chai S, Xu Q, Zeng L, Wang X, Jiang N, Sang M (2021). Resveratrol inhibits the progression of cervical cancer by suppressing the transcription and expression of HPV E6 and E7 genes. Int J Mol Med.

[34] Spurgeon ME, den Boon JA, Horswill M, Barthakur S, Forouzan O, Rader JS, Beebe DJ, Roopra A, Ahlquist P, Lambert PF (2017). Human papillomavirus oncogenes reprogram the cervical cancer microenvironment independently of and synergistically with estrogen. Proc Natl Acad Sci USA.

[35] Wu PL, Zeng C, Zhou YF, Yin L, Yu XL, Xue Q (2019). Farnesoid X receptor agonist GW4064 inhibits aromatase and ERβ expression in human endometriotic stromal cells. Reprod Sci.

[36] Journe F, Laurent G, Chaboteaux C, Nonclercq D, Durbecq V, Larsimont D, Body JJ (2008). Farnesol, a mevalonate pathway intermediate, stimulates MCF-7 breast cancer cell growth through Farnesoid-X-receptor-mediated estrogen receptor activation. Breast Cancer Res Treat.

[37] Kanao H, Enomoto T, Ueda Y, Fujita M, Nakashima R, Ueno Y, Miyatake T, Yoshizaki T, Buzard GS, Kimura T, Yoshino K, Murata Y (2004). Correlation between p14(ARF)/p16(INK4A) expression and HPV infection in uterine cervical cancer. Cancer Lett.

[38] Ghosh A, Ghosh S, Maiti GP, Sabbir MG, Alam N, Sikdar N, Roy B, Roychoudhury S, Panda CK (2009). SH3GL2 and CDKN2A/2B loci are independently altered in early dysplastic lesions of head and neck: correlation with HPV infection and tobacco habit. J Pathol.

[39] Weber HO, Samuel T, Rauch P, Funk JO (2002). Human p14(ARF)-mediated cell cycle arrest strictly depends on intact p53 signaling pathways. Oncogene.

[40] Llanos S, Clark PA, Rowe J, Peters G (2001). Stabilization of p53 by p14ARF without relocation of MDM2 to the nucleolus. Nat Cell Biol.

[41] Korgaonkar C, Zhao L, Modestou M, Quelle DE (2002). ARF function does not require p53 stabilization or Mdm2 relocalization. Mol Cell Biol.

[42] Liu H, Han YR, Zhan Y, Chen YF, Zhang YH, Yu H, Zhao TJ, Zhuo YZ (2007) A dynamical model on the network of p53-Mdm2 feedback loop regulated by p14/19ARF*.* In: Annual international conference of the IEEE Engineering in Medicine and Biology Society, 2007, pp 4235–4238 10.1109/IEMBS.2007.4353271 18002937

[43] Sohn D, Graupner V, Neise D, Essmann F, Schulze-Osthoff K, Jänicke RU (2009). Pifithrin-alpha protects against DNA damage-induced apoptosis downstream of mitochondria independent of p53. Cell Death Differ.

[44] Wang J, Ding S, Duan Z, Xie Q, Zhang T, Zhang X, Wang Y, Chen X, Zhuang H, Lu F (2016). Role of p14ARF-HDM2-p53 axis in SOX6-mediated tumor suppression. Oncogene.

[45] Zanjirband M, Edmondson RJ, Lunec J (2016). Pre-clinical efficacy and synergistic potential of the MDM2-p53 antagonists, Nutlin-3 and RG7388, as single agents and in combined treatment with cisplatin in ovarian cancer. OncoTarget.

