# Supplementary material for: Farnesoid X receptor functions in cervical cancer via the p14ARF-mouse double minute 2-p53 pathway
Source: Mol Biol Rep. 2022 Mar 28;49(5):3617–25. doi: 10.1007/s11033-022-07201-x (PMC9174312; doi:10.1007/s11033-022-07201-x)
Supplement: Supplementary file 1 — Supplementary file1 (DOCX 16 kb) [file 11033_2022_7201_MOESM1_ESM.docx]

Supplementary Table 1. Data of Tissue Slide

| Variable | Value |
| --- | --- |
| Total number  Normal cervical tissue  Cervical cancer  Stage  SⅠ  SⅡ  SⅢ  Based on the FIGO 2009 criteria. | 183  21  162  83  70  9 |

Supplementary Table 2. Data of Antibodies

| Antibodies | Companies | Item No. | Molecular weight (kDa) |
| --- | --- | --- | --- |
| β-actin  FXR  p14 ^ARF^  MDM2  p53  goat anti-rabbit IgG-HRP  goat anti-mouse IgG-HRP | Zsbio  Santa Cruz  Santa Cruz  Santa Cruz  Santa Cruz  Boster  Boster | TA-09  sc-13063  sc-53392  sc-965  sc-98  BA1054  BA1050 | 42  69  14  60  53 |

Supplementary Table 3. Primer sequences

| Genes | Sense (5’-3’) | Antisense (5’-3’) |
| --- | --- | --- |
| β-actin | TTGCTGATCCACATCTGCT | GACAGGATGCAGAAGGA |
| FXR | GATTGCTTTGCTGAAAGGGTC | CAGAATGCCCAGACGGAAG |

Supplementary Table 4. Data of *siRNA*

| Oligo | Sequences（5’-3’） | |
| --- | --- | --- |
| hs-p14^ARF^ | Sense strand | GGAGGCCGAUCCAGGUCAUdTdT |
|  | Anti-sense strand | AUGACCUGGAUCGGCCUCCdTdT |
| hs-MDM2 | Sense strand | GGAACUUGGUAGUAGUCAAdTdT |
|  | Anti-sense strand | UUGACUACUACCAAGUUCCdTdT |
| hs-TP53 | Sense strand | CCGGACGAUAUUGAACAAUdTdT |
|  | Anti-sense strand | AUUGUUCAAUAUCGUCCGGdTdT |
